# Automated sleep staging algorithms: have we reached the performance limit due to manual scoring?

**DOI:** 10.1093/sleep/zsac159

**Published:** 2022-07-22

**Authors:** Philip de Chazal, Diego R Mazzotti, Peter A Cistulli

**Affiliations:** Sleep Research Group, Charles Perkins Centre, The University of Sydney, Sydney, NSW, Australia; School of Biomedical Engineering, The University of Sydney, Sydney, NSW, Australia; Division of Medical Informatics, Department of Internal Medicine, University of Kansas Medical Center, Kansas City, KS, USA; Division of Pulmonary Critical Care and Sleep Medicine, Department of Internal Medicine, University of Kansas Medical Center, Kansas City, KS, USA; Sleep Research Group, Charles Perkins Centre, The University of Sydney, Sydney, NSW, Australia; Faculty of Medicine and Health, Northern Clinical School, The University of Sydney, Sydney, NSW, Australia; Department of Respiratory Medicine, Centre for Sleep Health and Research, Royal North Shore Hospital, Sydney, NSW, Australia

Sleep is essential for life, but its measurement has a brief history in evolutionary terms. The most widely accepted way of measuring sleep is via sleep stages derived from analysis of the electroencephalogram (EEG) which was first introduced by Loomis et al. [1] in 1937. Loomis et al. observed the continual changes of stages during sleep and identified five stages with the letters A-E with A containing trains of alpha waves, B sections of low voltage, C containing spindles, D containing spindles plus random potentials, and E containing random potentials only. Rapid eye movement (REM) sleep was first recognized in 1953 by Aserinsky and Kleitman [2]. In 1957 Dement and Kleitman recognized the cyclic nature of sleep stages [3] and simplified the sleep stages into four stages of non-REM sleep (S1–S4), a REM sleep stage, and a wake stage.

To standardize recording and scoring techniques and increase the equivalence of results between laboratories, the manual of Rechtschaffen and Kales (R&K) was introduced in 1968 [4]. In the R&K manual, a polysomnography (PSG) measurement is divided into 30-s epochs and a human scorer visually classifies each epoch into the five sleep stages and one wake stage of Dement et al. [3]. An extra stage was added to accommodate periods of movement. The intent of the R&K rules was to provide the minimum requirements for laboratories, but the manual became the gold standard for sleep studies for nearly 40 years. Over time criticisms were raised. The sleep stages were based on young healthy adult subjects, the epoch length of 30 s had no real physiological basis, two or more stages could be present in one epoch, transitioning rules between stages were undefined and there was no clinical difference between S3 and S4.

To address some of these criticisms and improve the interscorer agreement, the American Academy of Sleep Medicine (AASM) manual was released in 2007 [5]. The AASM manual uses 5 sleep stages: Wake (W), REM, and non-REM sleep (N1, N2, and N3). S3 and S4 from the R&K rules were merged into N3, and “movement time” was disregarded. Transition between sleep stages was more clearly defined, and in recognition of the development of digital recorders since the R&K rules, recommendations for sampling rates and filter settings for the PSG were given. The AASM also provides interscorer reliability (ISR) training as part of its scoring accreditation process. Technical staff members who score sleep studies for a sleep facility must participate in the ISR program with competency checked quarterly. Similar quality assurance programs exist across the world.

Partially due to the historical development of sleep analysis and because there is no true gold standard for sleep analysis, epoch-based manual sleep scoring is the current standard. Manual scoring can be performed by an individual scorer or a panel of scorers with the assumption that a panel of scorers is more consistent than a single scorer. Many studies have looked at the overall agreement between scorers. A very recent meta-analysis of 11 eligible studies [6] selected from 101 candidate studies concluded that the overall Cohen’s kappa for manual sleep scoring by individual scorers was 0.76, indicating substantial agreement. Analysis of the kappa values for individual sleep stages showed that agreement for wake and REM were substantial, agreements for stages N2 and N3 sleep were moderate, and for stage N1 sleep was fair. Studies comparing individual scorers to a panel of scorers are less common. An analysis of the agreement of individual scorers against a panel of six scorers from three centers in the United States scoring 70 PSGs showed that overall kappa was 0.57 indicating moderate agreement [7].

Considerable progress has been made with machine-based sleep stage scoring and current evidence suggests that the performance of machine-based scoring is comparable to human scoring. A review of the state-of-the-art machine-based scorers in 2019 revealed that the overall agreement of machine-based sleeping staging against individual scorers was a kappa value in the range 0.73–0.86 [8]. An analysis of performance on individual sleep stages revealed worst performance on N1 and best on N3, W, or REM reflecting the same trends as human scorers. One of these studies compared performance against a panel of scorers obtaining a kappa of 0.76 [7].

The development of machine-based scoring sleep systems requires a training stage whereby sleep stages from human scorers along with PSG signals are provided to machine learning algorithms and the system uses these manually labeled sleep stages to learn associations between the signals and the sleep stages. Given that human and machine scoring achieve comparable agreement the obvious question arises as to whether machines can provide better performance than they currently do. In this issue of Sleep, van Gorp et al. [9] provide a theoretical framework from the statistics and the machine learning community to facilitate discussion on the uncertainty in sleep staging. They introduce two variants: aleatoric and epistemic uncertainty. Aleatoric uncertainty arises from the random nature of data and their measurements. Epistemic uncertainty arises from a lack of knowledge about the data or the optimal model. Aleatoric uncertainty is inherent to a specific measurement setup and cannot be reduced, whereas epistemic uncertainty may be reduced through additional training and/or further data collection. This raises the question of whether the limit in performance that machine-based scorers have reached is an artifact of the determination of ground truth. The uncertainty on ground truth and its impact on machine-based scoring systems is not limited to sleep stages. It equally applies to other measurements in our field, such as the apnea–hypopnea index.

One path forward may be through the wider use of panel-based scoring but, given the high workload for panel-based scoring, our field must test the assumption that panel-based scoring is actually more consistent than individual scoring. Another approach is to consider observing sleep as a continuous, non-discrete dynamic phenomenon. These definitions of “continuous sleep” have been recently proposed using both machine learning, with the description of hypnodensity [7] as well as using expert knowledge about EEG dynamics during sleep to calculate the odds ratio product (ORP) as a continuous measure of sleep depth [10–12]. The hypnodensity expresses sleep in terms of a probability distribution in 5-second epochs, providing more granularity. Epochs associated with higher inter-rater variability (such as N1) typically result in higher model variance. The ORP, as well as its distribution across the night, have been recently demonstrated to capture clinically relevant information, including associations with sleep disorders such as insomnia, OSA, sleepiness, and perceived poor sleep quality [11–13]. However, the utility of ORP to characterize REM sleep dynamics is still underexplored. An exciting future direction is the use of semi-supervised and unsupervised learning to fully harness the potential of the electrophysiological data available in describing sleep. Could the lower inter-rater variability in N1 and higher model variance in machine learning methods be explained by the existence of well-defined sub-stages or sleep transition states? If these states truly exist and prove to be clinically significant, it is possible that our current ideas on ground truth may change. From our perspective, this will fully leverage the synergistic contributions of humans and machines towards the understanding the mysteries of sleep.
